# Favorable Neurological Recovery After Prolonged Absence of Antegrade Cerebral Blood Flow During Congenital Heart Surgery: A Case Suggesting the Role of Retrograde Perfusion

**DOI:** 10.7759/cureus.84002

**Published:** 2025-05-13

**Authors:** Koji Hoshino, Sora Takeuchi, Kazuhiro Urabe, Gou Matsuzawa, Yuji Morimoto

**Affiliations:** 1 Department of Anesthesiology, Hokkaido University Hospital, Sapporo, JPN

**Keywords:** cardiac arrest, cardiopulmonary resuscitation, congenital heart disease (chd), near-infrared spectroscopy, retrograde cerebral perfusion

## Abstract

Intraoperative cardiac arrest during pediatric congenital heart disease surgery is rare but associated with high mortality and poor neurological outcomes. While cerebral perfusion during cardiopulmonary resuscitation is typically antegrade, retrograde cerebral flow has not been reported.

We report a case of a 12-year-old girl undergoing pulmonary artery debanding for congenitally corrected transposition of the great arteries. Massive hemorrhage from pulmonary artery injury led to cardiac arrest, during which antegrade cerebral flow was likely absent for 15 minutes. Nevertheless, the patient recovered without neurological sequelae. Time-domain near-infrared spectroscopy showed increased deoxygenated hemoglobin and preserved total hemoglobin, suggesting cerebral perfusion via retrograde venous flow. Central venous pressure was maintained at 15-25 mmHg through rapid transfusion.

This case suggests that retrograde cerebral perfusion may occur during cardiac arrest when central venous pressure is adequately maintained, potentially contributing to favorable neurological outcomes even in the absence of antegrade flow.

## Introduction

The incidence of intraoperative cardiac arrest in pediatric congenital heart disease (CHD) surgery has been reported to be 0.38%, based on a retrospective study analyzing approximately 90,000 cases from the Society of Thoracic Surgeons Congenital Heart Surgery Database [1]. According to their report, intraoperative cardiac arrest increases mortality and raises the risk of neurological sequelae. A study by Matos et al., which analyzed approximately 3,400 cases of in-hospital pediatric cardiac arrest, found that the proportion of patients achieving favorable neurological outcomes was only 19% [2]. Furthermore, for each additional minute of cardiopulmonary resuscitation (CPR), the survival rate decreased by 2.1%, and the likelihood of a favorable neurological outcome declined by 1.2% per minute. Therefore, achieving favorable neurological outcomes in intraoperative cardiac arrest among pediatric CHD patients remains a significant challenge.

Aortic blood flow during CPR is generally considered antegrade [3], and there have been no reports describing the involvement of retrograde cerebral blood flow. Here, we present a case of intraoperative cardiac arrest due to massive hemorrhage following pulmonary artery injury in a pediatric CHD patient, in which retrograde cerebral blood flow may have contributed to a favorable neurological outcome. Near-infrared spectroscopy (NIRS), a widely used non-invasive method for monitoring cerebral oxygenation during cardiac surgery, played a key role in this case by providing real-time physiological data that supported the hypothesis of retrograde cerebral perfusion under extreme circulatory conditions.

## Case presentation

A 12-year-old girl (147 cm, 38.2 kg) was diagnosed with congenitally corrected transposition of the great arteries at birth and underwent pulmonary artery banding during infancy. Due to the progression of heart failure associated with increased left ventricular pressure, she was scheduled for pulmonary artery debanding surgery. Anesthesia was induced via a 24-gauge peripheral intravenous line in the left upper limb using midazolam, fentanyl, and rocuronium, followed by tracheal intubation. Anesthesia was maintained with continuous infusion of propofol and remifentanil, along with intermittent administration of fentanyl and rocuronium. A radial arterial line was placed in the left arm, and an additional 20-gauge peripheral venous line was inserted into the right upper limb. A central venous catheter was placed in the right internal jugular vein. Bispectral index (BIS; Covidien Japan Co., Ltd., Tokyo, Japan) and t-NIRS1 sensors (Hamamatsu Photonics Co., Ltd., Hamamatsu, Japan) were applied bilaterally to the forehead. The patient's vital signs remained stable from anesthesia induction to thoracotomy.

**Figure 1:**
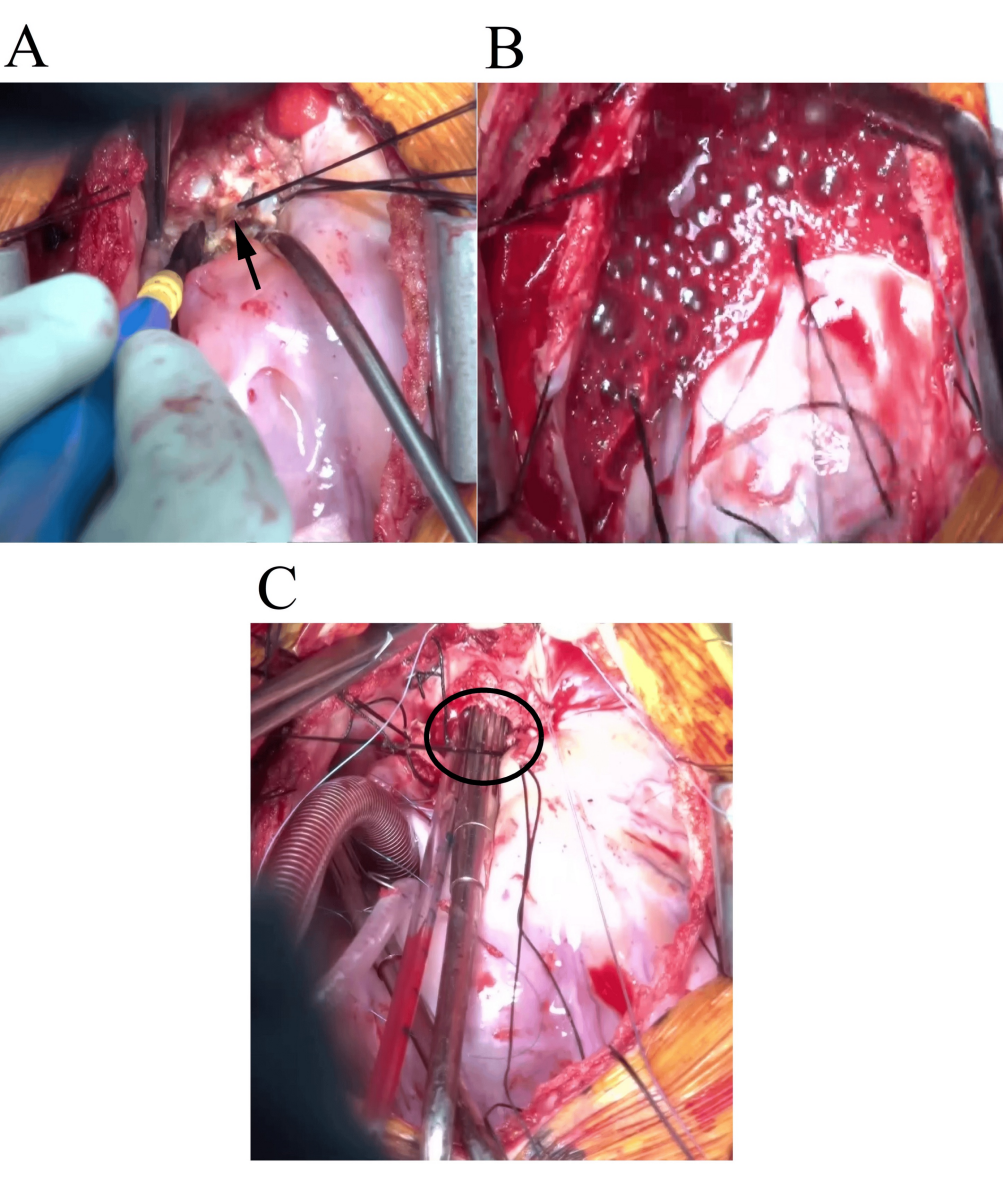
Intraoperative images illustrating the progression of pulmonary artery hemorrhage. (A) Detaching the pulmonary artery banding tape (arrow) immediately prior to the onset of bleeding. (B) Uncontrollable hemorrhage from the anterior wall of the main pulmonary artery. (C) After the initiation of cardiopulmonary bypass, the injury on the anterior wall of the main pulmonary artery was directly visualized, showing that the defect had enlarged to the extent that a surgical suction tip could be inserted into the injury site (circled area).

**Figure 2:**
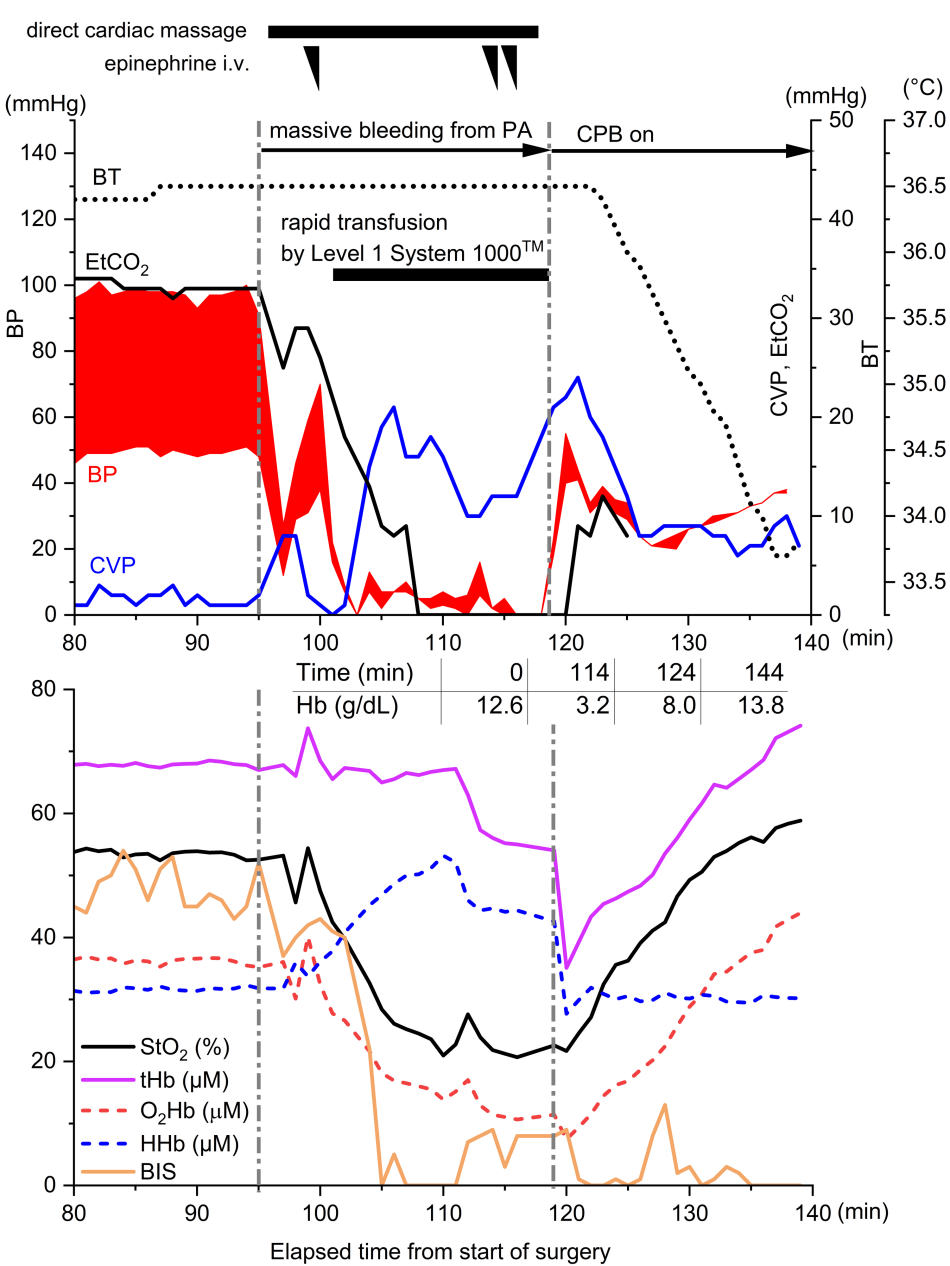
Time course of arterial blood pressure, central venous pressure, end-tidal carbon dioxide, and core temperature before and after massive pulmonary artery hemorrhage (upper panel); trends in t-NIRS1 parameters (StO2, O₂Hb, HHb, tHb) and bispectral index (lower panel); and time course of systemic hemoglobin concentrations from arterial blood gas analysis (middle table). The t-NIRS1 data represent the average values from bilateral sensors. Although pulse pressure dropped to zero for 15 minutes following pulmonary artery injury, the decrease in tHb measured by t-NIRS1 remained minimal. CVP: central venous pressure; BP: blood pressure; EtCO_2_: end-tidal carbon dioxide; BT: body temperature; PA: pulmonary artery; CPB: cardiopulmonary bypass; Hb: hemoglobin; StO_2_: tissue oxygen saturation; tHb: total hemoglobin; O_2_Hb: oxygenated hemoglobin; HHb: deoxygenated hemoglobin; BIS: bispectral index.

Ninety-five minutes after the start of surgery, pulmonary artery injury occurred during detaching of the pulmonary artery banding site (Figure 1A), leading to massive hemorrhage and cardiac arrest (Figure 2, upper panel). Immediate direct cardiac massage was initiated; however, due to the expansion of the injury, bleeding control became unmanageable (Figure 1B). While the surgeons prepared for emergency cardiopulmonary bypass (CPB), the anesthesiologists initiated head cooling with cooling packs and administered a total of approximately 1,400 mL of red blood cell (RBC) products via rapid transfusion using the LEVEL 1 System 1000 (Smiths Medical Japan Co., Ltd., Tokyo, Japan) and manually infused approximately 750 mL of 5% albumin solution via the central and peripheral venous lines during the cardiac arrest period. Central venous pressure (CVP) was maintained at 15-20 mmHg. Despite multiple 1 mg doses of epinephrine, there was no response, and the arterial blood pressure waveform showed an almost zero pulse pressure for approximately 15 minutes. End-tidal carbon dioxide (EtCO₂) became unmeasurable, suggesting an absence of antegrade blood flow.

Meanwhile, intra-arrest monitoring with the t-NIRS1 system demonstrated a decrease in tissue oxygen saturation (StO₂); however, total hemoglobin (tHb) concentration showed only a mild decline, while deoxygenated hemoglobin (HHb) levels increased and oxygenated hemoglobin (O₂Hb) levels decreased (Figure 2, lower panel). These findings suggested that cerebral blood volume was maintained by venous circulation.

Twenty-three minutes after pulmonary artery injury, CPB was initiated via suction-assisted venous drainage and arterial perfusion through the right common femoral artery. Approximately 60% of the target CPB flow was achieved initially. Subsequently, a venous drainage cannula was placed in the right atrium, allowing total flow to be established, and surgery continued. At this point, the injury on the anterior wall of the main pulmonary artery had enlarged to approximately 10 mm (Figure 1C). During CPB, mild hypothermia (34°C) was maintained. Since the initiation of CPB, StO₂ remained consistently above 50%; however, BIS values showed only a gradual increase despite adjustments in anesthetic dosage and remained at 25 at the end of the operation. The CPB duration was two hours and eight minutes, and the total operative time was six hours and 27 minutes. The total intraoperative blood loss was 3,800 mL.

Postoperatively, targeted temperature management at 36°C was maintained for 48 hours in the intensive care unit (ICU). During this period, given the persistently low BIS values, no pharmacologic neuroprotective interventions were administered beyond temperature management, and sedation was maintained with continuous infusions of dexmedetomidine and midazolam. Rewarming was completed on postoperative day three, and upon awakening from anesthesia, the patient was alert, hemodynamically stable, and successfully extubated. She was transferred out of the ICU on postoperative day four. During the ICU stay, the patient tested positive for delirium using the Confusion Assessment Method for the ICU (CAM-ICU), and a delirious state persisted. On postoperative day five, mild higher cognitive dysfunction was observed, including errors in simple calculations; however, by day eight, the patient had recovered sufficiently to complete school assignments, and she was discharged on postoperative day 16. Postoperative head computed tomography showed no evidence of structural abnormalities.

## Discussion

We experienced a case in which antegrade cerebral blood flow was presumed to have been interrupted for approximately 15 minutes due to massive hemorrhage from the pulmonary artery, yet the patient recovered without neurological sequelae.

The t-NIRS1 system utilizes time-domain near-infrared spectroscopy (TD-NIRS), which emits short-pulse light to determine the absolute values of absorption coefficients. Consequently, t-NIRS1 allows for the absolute measurement of StO2, O₂Hb, HHb, and tHb. Compared to other NIRS devices using different algorithms, t-NIRS1 has been reported to offer the most accurate and reproducible measurements of cerebral oxygenation, particularly in pediatric patients, due to its reduced susceptibility to extracranial contamination and absence of ceiling effects [4]. Ohmae et al. have theoretically described the relationship between tHb measured by TD-NIRS and cerebral blood volume (CBV) using the following equation:

CBV (cc/100g) = $( tHb \times MWHb ) / ( Hb \times \eta \times \rho \times 100,000 )$

Where MWHb is the molecular weight of hemoglobin (64,500), Hb represents the capillary hemoglobin concentration in the brain (g/dL), η is the cerebral-to-large-vessel hematocrit ratio (0.85), and ρ is the density of cerebral tissue (1.04 g/mL) [5].

In this case, during cardiac arrest, a decrease in O₂Hb and an increase in HHb were observed, but the decline in tHb was minimal. Based on the equation above, this suggests that CBV was likely maintained. Additionally, given that the patient exhibited nearly zero pulse pressure and undetectable EtCO₂ during cardiac arrest, it is evident that antegrade cerebral blood flow was essentially absent. The combination of these findings and the favorable neurological outcome raises the possibility that retrograde blood flow may have been present.

From a theoretical standpoint, in cases of massive hemorrhage, the denominator (Hb) in the CBV equation should have decreased. However, as the numerator (tHb) remained relatively unchanged, CBV should have significantly increased, which creates a theoretical contradiction. This discrepancy may be explained by using rapid transfusion primarily with RBC products with a high hematocrit, which could have reached the brain via retrograde flow, thereby maintaining capillary hemoglobin concentration.

Even after pulse pressure became zero, the rapid transfusion of RBCs maintained CVP at 15-25 mmHg. In this patient, who had undergone pulmonary artery banding, reduced outflow toward the pulmonary artery likely contributed to maintaining venous pressure. Furthermore, a transesophageal echocardiography study has reported the presence of retrograde intracardiac flow during chest compressions [6]. Although direct cardiac massage was performed in this case, it may have similarly generated flow from the right ventricle to the right atrium and the superior vena cava.

In aortic surgery, retrograde cerebral perfusion using CPB is sometimes employed as a neuroprotective strategy [7]. An experimental study in dogs demonstrated that a perfusion pressure of approximately 20 mmHg was optimal in terms of cerebral metabolism [8]. Furthermore, Usui et al. reported that within the range of 15-25 mmHg, a linear relationship between jugular venous pressure and retrograde cerebral perfusion was observed [9]. In this case, CVP was maintained between 15 and 25 mmHg during cardiac arrest, and with an arterial pressure of zero, conditions may have been favorable for retrograde perfusion. Interestingly, immediately after the initiation of emergency CPB, tHb paradoxically decreased at that point, which may have been due to hemodilution but could also suggest that antegrade cerebral blood flow was initially insufficient due to competition with pre-existing retrograde flow.

The combined use of t-NIRS1 and BIS has been reported to be useful in detecting cerebral hypoxia during open-heart surgery [10]. According to Sugiura et al., BIS and tHb typically exhibit parallel trends intraoperatively [10]. However, in this case, BIS did not increase even after the initiation of CPB, showing a discrepancy with the tHb values. Normally, when cerebral blood flow falls below 22 mL/100 g/min, electrical activity first declines, leading to a functionally reversible state known as the "penumbra" [11]. This suggests that low retrograde perfusion during CPR may have induced the development of a penumbra region in this patient. In fact, StO₂ remained below 30% during the period of zero pulse pressure. Unlike CPB-based retrograde cerebral perfusion, the cerebral perfusion in this case likely consisted of deoxygenated blood, which would account for lower StO₂ levels. However, as seen in conventional CPR, the presence of even deoxygenated blood flow can contribute to improved neurological outcomes [12], highlighting the importance of maintaining cerebral perfusion regardless of oxygenation.

Nevertheless, there is no direct evidence confirming the presence of retrograde cerebral blood flow in this case. Caution must be taken when extrapolating these physiological observations to broader clinical scenarios. First, while CBV could be estimated, transcranial Doppler was not used to measure cerebral blood flow, preventing direct confirmation. Additionally, the study by Matos et al. indicated that among pediatric in-hospital cardiac arrests, those occurring in surgical cardiac patients had the best neurological outcomes [2]. Thus, it is possible that the young age of this patient and the rapid establishment of emergency CPB, limiting the cardiac arrest duration to 15 minutes, contributed to the favorable outcome. However, given that antegrade cerebral blood flow was nearly absent for 15 minutes, the rapid recovery and early discharge of this patient may not be fully explained by age alone. Finally, previous reports have documented cases where cerebral tissue oxygenation remained high despite an absence of blood flow [13], suggesting the possibility of erroneous NIRS measurements. However, in this case, the normalization of values following CPB initiation supports the reliability of the t-NIRS1 data.

## Conclusions

This case highlights the potential importance of maintaining cerebral perfusion even in the absence of antegrade blood flow during cardiac arrest caused by severe pulmonary artery bleeding. Although the patient experienced a prolonged period without measurable pulse pressure, favorable neurological recovery was achieved. Continuous monitoring using t-NIRS1 suggested preserved cerebral blood volume, and central venous pressure remained elevated through rapid transfusion. These findings raise the possibility that retrograde cerebral perfusion may have contributed to neuroprotection under extreme circulatory conditions. While direct evidence of retrograde flow is lacking, this case underscores the need to consider strategies that maintain venous pressure and cerebral perfusion during similar intraoperative emergencies. Moreover, this case may illustrate the potential utility of TD-NIRS in monitoring cerebral physiology during profound circulatory compromise. In light of the growing interest in intraoperative neuromonitoring, further studies are warranted to validate the clinical applicability and prognostic value of this technology.

## References

[1] Brown ML, Staffa SJ, Adams PS (2024). Intraoperative cardiac arrest in patients undergoing congenital cardiac surgery. JTCVS Open.

[2] Matos RI, Watson RS, Nadkarni VM (2013). Duration of cardiopulmonary resuscitation and illness category impact survival and neurologic outcomes for in-hospital pediatric cardiac arrests. Circulation.

[3] Andreka P, Frenneaux MP (2006). Haemodynamics of cardiac arrest and resuscitation. Curr Opin Crit Care.

[4] Kubo Y, Itosu Y, Kubo T, Saito H, Okada K, Ito YM, Morimoto Y (2024). Cerebral oxygenation saturation in childhood: difference by age and comparison of two cerebral oximetry algorithms. J Clin Monit Comput.

[5] Ohmae E, Ouchi Y, Oda M (2006). Cerebral hemodynamics evaluation by near-infrared time-resolved spectroscopy: correlation with simultaneous positron emission tomography measurements. Neuroimage.

[6] Kim H, Hwang SO, Lee CC (2008). Direction of blood flow from the left ventricle during cardiopulmonary resuscitation in humans—its implications for mechanism of blood flow. Am Heart J.

[7] Ueda Y (2013). A reappraisal of retrograde cerebral perfusion. Ann Cardiothorac Surg.

[8] Nojima T, Magara T, Nakajima Y, Waterida S, Onoe M, Sugita T, Mori A (1994). Optimal perfusion pressure for experimental retrograde cerebral perfusion. J Card Surg.

[9] Usui A, Oohara K, Liu TL, Murase M, Tanaka M, Takeuchi E, Abe T (1994). Determination of optimum retrograde cerebral perfusion conditions. J Thorac Cardiovasc Surg.

[10] Sugiura A, Torii K, Tsutsumi H (2021). Effective method of monitoring cerebral tissue oxygen saturation in cardiac surgery patients by combined use of tNIRS-1 and bispectral index. Sci Rep.

[11] del Zoppo GJ, Sharp FR, Heiss WD, Albers GW (2011). Heterogeneity in the penumbra. J Cereb Blood Flow Metab.

[12] Cabrini L, Biondi-Zoccai G, Landoni G (2010). Bystander-initiated chest compression-only CPR is better than standard CPR in out-of-hospital cardiac arrest. HSR Proc Intensive Care Cardiovasc Anesth.

[13] Maillard J, Sologashvili T, Diaper J, Licker MJ, Keli Barcelos G (2019). A case of persistence of normal tissue oxygenation monitored by near-infrared spectroscopy (NIRS) values despite prolonged perioperative cardiac arrest. Am J Case Rep.

